# The effect of automated fiber placement process parameters on interlaminar shear strength of uncured prepreg bonded samples

**DOI:** 10.1177/00219983241313280

**Published:** 2025-01-11

**Authors:** Meisam Kheradpisheh, Amir Hafez Yas, Mehdi Hojjati

**Affiliations:** Department of Mechanical, Industrial and Aerospace Engineering, Concordia Center for Composites (CONCOM), Montreal, QC, Canada

**Keywords:** Inter-laminar shear strength, automated fiber placement process parameters, single lap joints, uncured prepreg, cohesive zone model

## Abstract

The effects of automated fiber placement (AFP) parameters on the inter-laminar bonding between the uncured thermoset prepreg tapes were investigated using a systematic series of experiments and FE analysis. The goal was to optimize inter-laminar bonding during the AFP lay-up process and provide a model for the interlayer bonding of uncured prepreg tapes during this process. The shear strength of the interfacial bonding plays a pivotal role in the formation of planar and non-planar deformations during the automated fiber placement (AFP) process. The quality of this bonding has a significant effect on the quality of the manufactured parts. Besides, the bonding strength is interconnected with various AFP process parameters including compaction roller, feed rate, temperature, and dwell time. Hence, a systematic series of experimental studies are conducted to investigate how changes in process parameters affect the shear strength of single lap joint (SLJ) specimens produced under various process conditions. To fabricate the single-lap joint samples under different conditions, an in-house setup was developed to simulate the AFP process allowing us to control compaction force, feed rate, temperature, and dwell time during the process. The experimental results of the single lap joints indicate that the shear strength of the bonded prepreg tows is significantly influenced by the interaction among the process parameters rather than by their individual, isolated effects. Moreover, the responses of prepreg SLJs are simulated using the FE method. Through the comparison of numerical and experimental results, it will be clearly shown that the developed FE framework can act as a reliable approach for modeling the bonding layer between prepreg tapes.

## Introduction

The automated fiber placement (AFP) process is an advanced manufacturing technology that is widely used in industries such as automotive, and aerospace, thanks to the high productivity, cost-effectiveness, precise fiber placement, and low wastage. Besides, carbon fiber thermoset prepreg tapes are used as a raw material in the thermoset AFP process proves to be a viable substitute for the traditional materials by offering a high stiffness and strength, corrosion resistance, and chemical resistivity.

**Figure 1.**
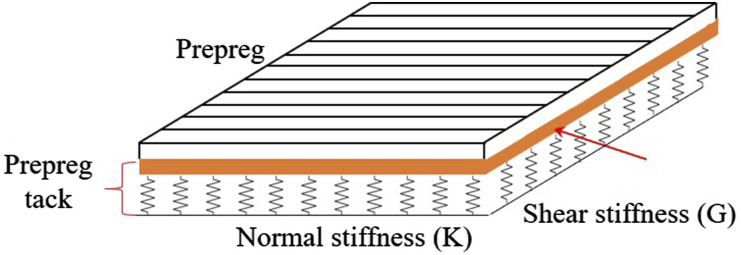
A schematic of prepreg tape model as a composite plate on Pasternak foundation.

In the AFP process for creating a composite part, the initial step involves placing the multiple adjusted prepreg tows to create a course. Then, these courses are positioned in a predetermined sequence to form a ply. The progressive layering of plies, with each ply placed next to and on top of each other, leading to the formation of laminate. This manufacturing process is performed using the robotic head of the AFP machine that precisely controls the placement of the prepreg materials on the substrate. The head of the machine is mounted on a six degrees of freedom system which provides accurate movement in various directions. This machine’s head is equipped with a guidance system that directs the prepreg tow by providing proper tension from the spool to the tip of the head where both the compaction roller and the heat source are positioned. In this stage, the pressure and heat are applied to the prepreg tapes to adhere them onto the substrate. The quality of the product manufactured by the AFP robot is notably influenced by their adhesion to the substrate, the adhesion between the prepreg tape and substrate is also known as prepreg tack. Insufficient adhesion or prepreg tack may lead to the formation of defects such as gap, overlap, in-plane, and out-of-plane buckling during the AFP process.^1–3^ To determine the effect of prepreg tack on defect formation, several studies have developed analytical models that investigate the adhesion between the prepreg and the substrate. In these models, the prepreg is treated as a plate supported by an elastic foundation, which represents the prepreg tack. The elastic foundation is modeled using normal and shear springs, as depicted in Figure 1. By deriving the equilibrium equations, these models show that in-plane and out-of-plane defects are related to the shear and normal stiffness of the elastic foundation, and higher values of *K* and *G* prevent defect formation during the lay-up process. Since such defects persist after curing and diminish the quality of the final part, minimizing and eliminating them is essential for enhancing part quality (for more details see Refs. 4–6). Besides, a source of gaps and overlaps in the automated manufacturing of composites is the misalignment between adjacent tows caused by placement head positioning errors. The combination of computer errors with the increase in the width of the prepreg can increase the generation of overlap and gaps in the manufacturing process. This occurs because AFP software accounts for a specific tape width during lay-up and any deviation—whether an increase or decrease—from that specified width can lead to the formation of gaps and overlaps.

The quality of the prepreg’s stickiness to the substrate can be attributed to the material’s aging which is investigated in Refs. 7–10. The work in Refs. 7 studied the normal stickiness of prepreg tape under different aging conditions. Investigating the impact of humidity, time, and temperature on prepreg tack in the normal direction was carried out through a probe test. In Refs. 8, the quality of the prepregs was investigated under different environmental conditions. Various experimental methods, including differential scanning calorimetry (DSC), thermal analysis (TA), and infrared spectroscopy (IR) were employed to evaluate the performance of prepreg material during the storage time. Smith et al.^9,10^ examined the evaluation of the prepreg tack over a span of up to 375 days of environmental aging. The authors in Refs. 11 studied the effect of room-temperature aging on the glass transition temperature and degree of cure using the DSC test. The results show that aging reduces the performance of the prepreg materials.

In addition to aging, the stickiness of the prepreg tapes can still be influenced by various AFP process parameters, including pressure, dwell time, temperature, and feeding rate.^12^ As mentioned earlier, the inadequate stickiness of prepreg to the substrate may lead to the occurrence of defects at prepreg tape during the lay-up process. For example, insufficient tackiness in the steering process, which is the placing of tow in a curvilinear path, forms in-plane and out-of-plane buckling defects in the inner edge of the tape. As previously mentioned, the insufficient adhesion of the prepreg to the substrate can result in the occurrence of defects in the prepreg tape during the lay-up process. For instance, insufficient tackiness in the steering process, where the tow is placed in a curvilinear path, can lead to in-plane and out-of-plane deformations in the inner edge of the tape.^5,13,14^ Therefore, investigating the impact of the process parameters on the stickiness of the prepreg is significant in enhancing its adhesion and minimizing the formation of defects. In this context, the authors^15,16^ designed a novel peel test apparatus to quantify the prepreg tack in the normal direction (90°). This test setup facilitated the simulation of the AFP process, allowing them to record peel resistance and study the effect of feed rate and pressure on the peel resistance of the prepreg materials. Budelman et al.^2^ employed rheometer equipment to measure the stickiness of the prepreg in the normal direction using the probe tack test. The temperature was adjusted using a convection oven, while the pressure, dwell time (compaction period), and debonding rate were controlled through the dynamic probe fixture of the rheometer. They showed that around 40°C is the optimal temperature for achieving the highest tack energy. Furthermore, their results indicated that an increase in the debonding rate led to a rise in the maximum tack stress. In Refs. 12 the effect of process parameters, including temperature, pressure, and feed rate, on prepreg tack was assessed through peel-off tests. They utilized the Taguchi method to optimize and streamline the experimental design, reducing the number of required trials.

In addition to examining the influence of process parameters on stickiness in the normal direction, it is crucial to investigate their effects on stickiness in the shear direction. This consideration is essential due to the rolling of the roller and the steering process of the prepreg, generating an in-plane force at the prepreg tape and inducing movement on the substrate in the shear direction.^5,17^ The effect of the shear modulus on the formation of wrinkle defects in the AFP process was demonstrated through a mathematical approach in Refs. 5. In their buckling model developed for wrinkle formation, they accounted for the impact of the shear layer by incorporating it into the elastic foundation. The in-plane shear response of uncured prepregs was investigated in the work presented by^17^ using a 10° off-axis tensile test, then, a viscoelastic model was presented for modeling the calculated in-plane shear modulus in Refs. 18. The authors in Refs. 19 carried out an experimental study to find the inter-laminar shear behavior of the uncured prepreg tapes by means of ±45° tensile test. In addition, the impact of the temperature, the number of layers, and the tensile rate on inter-laminar shear behavior were presented. Inter-laminar friction properties of uncured thermoset prepregs and dry prepregs through a novel tensile friction test were studied in Refs. 20,21. The work in Refs. 22 investigated the effect of AFP process parameters on the mechanical properties of thermoplastic prepregs. The research employed four-point bending and single-lap joint tests to assess these mechanical properties under different process conditions.

Previous research on prepreg tack (adhesion between prepreg and substrate) has largely concentrated on their normal separation. However, the analytical models suggest that inter-layer shear properties are critical for minimizing defects in thermoset AFP manufacturing (see Figure 1. Therefore, optimizing AFP process parameters to enhance the bonding of prepreg tape is crucial for achieving high-quality manufactured structures. This study aims to extensively investigate the inter-layer shear behavior of uncured thermoset prepreg tapes under various process parameters including temperature, compaction roller, lay-up speed, and contact time. For this purpose, single-lap joint (SLJ) prepreg samples were fabricated and tested under these varying conditions to assess shear strength and determine the optimal processing conditions. In this context, a cohesive zone model-based FE analysis was developed to simulate SLJ sample responses, providing stress distributions and bonding layer behavior under various conditions. Moreover, microscopic and geometric analyses were also performed to provide a deeper understanding of shear behavior in bonded uncured prepreg tapes.

## Materials and manufacturing procedure

The material used for the fabrication of the single lap joint samples was an uncured thermoset unidirectional carbon fiber/epoxy prepreg tape (CYCOM 977-2) supplied by Bombardier Inc. in Canada. The thermoset prepreg tapes have a 6.35 mm width and 0.17 mm thickness and contain 60 % fiber content by volume.

### Sample preparation

**Figure 2.**
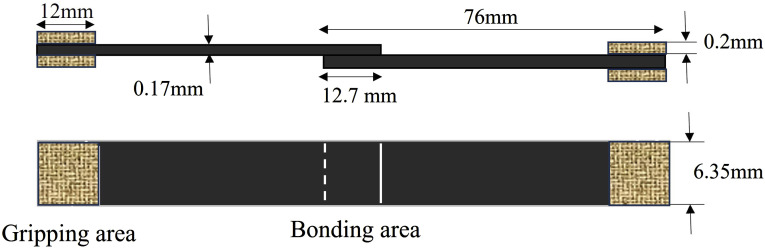
Schematic of single-lap joint unidirectional prepreg specimen with dimensions.

In the current work, due to the absence of an ASTM standard specifically designed for measuring the shear strength of prepreg tapes, ASTM D5868 was utilized for the preparation of the SLJ samples, with some adjustments made to the specimen dimensions. The schematic shown in Figure 2 provides a visual representation of the configuration of the specimens. Since the matrix of the uncured prepreg creates a bond between the two pieces of prepreg, there is no need for additional adhesive (see Figure 2).

**Figure 3.**
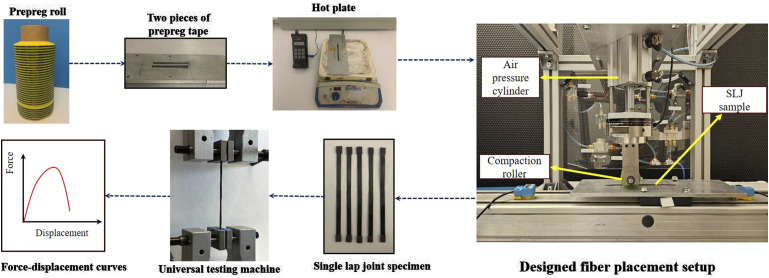
The manufacturing procedure of the single lap joint specimen.

The creation procedure of single lap joint samples begins with the cutting of two pieces of prepreg from a prepreg roll that has been kept at room temperature for a duration of 2 hours. These uncured prepreg pieces are then positioned on an aluminum substrate, ensuring a 12.7 mm (0.5 Inch) overlap. Then, a hot plate is used to control the temperature of the SLJ samples. In the next step, a designed automated fiber placement (AFP) setup is utilized which is capable of changing pressure and feed rate values during the sample preparation. As shown in Figure 3, the AFP setup utilizes an air cylinder connected to a load cell to precisely control the force applied to the compaction roller. The compaction roller is held by an air jack attached to a steel bracket providing stability and facilitating vertical motion. For the horizontal movement, the system is equipped with a ball screw linear guide, which allows the aluminum plate to move at variable speeds beneath the compaction roller. After applying pressure at different velocities, the tabs are attached to the samples in the gripping area and subsequently, a universal testing machine is employed to conduct quasi-static single-lap shear tests for assessing the shear strength of individual specimens. During these tests, the SLJ samples were subjected to a constant displacement rate of 1.5 mm/min. To guarantee accuracy and reproducibility in the test outcomes, four tests were conducted for every sample. Figure 3 shows the whole manufacturing procedure of single-lap joint bonded with prepreg adherents.

**Figure 4.**
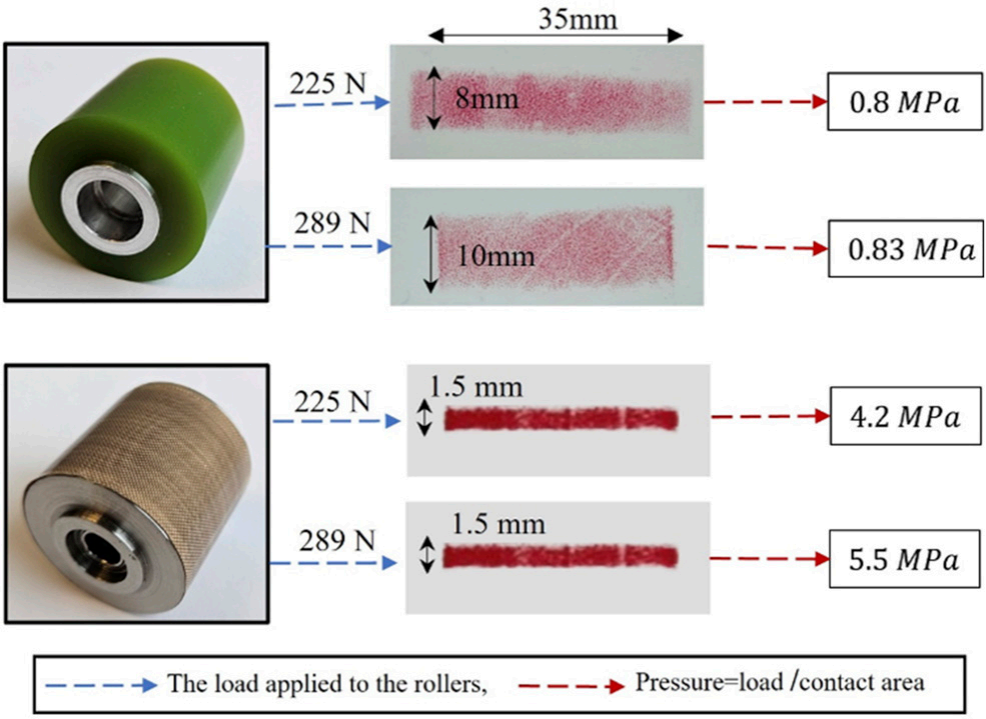
Two different compaction rollers and the values of four different levels of pressure were used in the preparation of SLJ samples.

Since this research investigates the effects of four process parameters: temperature, pressure, feed rate, and dwell time on the inter-laminar shear strength of the uncured prepreg tape, with regard to the experiment’s limitations, we systematically considered different levels for each parameter to explore their effects:

(i) For the pressure parameters: Two common compaction rollers were employed: (1) a polyurethane roller (PUR) with 60 durometer hardness and (2) a stainless-steel roller (SSR). Each roller was subjected to two distinct loads: 225 N and 289 N, representative of typical loads during the thermoset AFP process. This resulted in four different pressure levels, calculated by dividing the applied load by the contact area created by the roller under the applied load. As a result, the PUR roller generated pressures of 0.8 MPa and 0.83 MPa, while the SSR roller produced pressures of 4.2 MPa and 5.5 MPa. Figure 4 shows the rollers and their contact area traced on pressure film and the values of pressure.
(ii) For the Temperature Parameter: Due to measurement limitations and the low operational temperature, two levels were considered for the temperature: (1) room temperature, approximately 25 degrees Celsius, and (2) a higher temperature of around 45 degrees Celsius, which corresponds to the point where the adhesive bonding between prepreg and substrate is maximized.^2^
(iii) For the feeding rate: Three different speeds including 15 mm/s, 35 mm/s, and 55 mm/s were selected for the feeding rate parameter.
(iv) For the dwell time (compaction period): As mentioned in the last paragraph of the introduction section, we define dwell time as the amount of time during which each point of tape is beneath the roller. Thus, it is a function of both contact area and pressure and can be calculated by dividing the length of the contact area in the direction of movement by the feeding rate, as expressed in the following equation:

(1) $$Dwell\;time=\frac{Contact\;length}{Feed\;rate}$$

For a better understanding of the experimental conditions, the process parameters, compaction roller types, and levels of each parameter are summarized in Table 1

**Table 1.** Process parameters’ levels for fabrication of single-lap joint samples.

Parameters	Type of roller		Levels
Pressure	PUR		0.8 MPa and 0.83 MPa
	SSR		4.2 MPa and 5.5 MPa
Feed rate	For both roller		15, 35, 55 and 80 mm/s
Dwell time	PUR with pressure of	0.8 MPa	0.53, 23, 14, 0.1 s
		0.83 MPa	0.67, 28, 18, 0.15 s
	SSR with pressure of	4.2 MPa	0.1, 0.04, 0.02, 0.018 s
		5.5 MPa	0.1, 0.04, 0.02, 0.018 s
Temperature	For both roller		25 and 45 degree Celsius

## Experimental results and discussion

In this section, the force-displacement curves are plotted for specimens manufactured under various process conditions. Moreover, the influence of process parameters on the shear strength of the single lap joint samples is examined.

### Pressure effect

**Figure 5.**
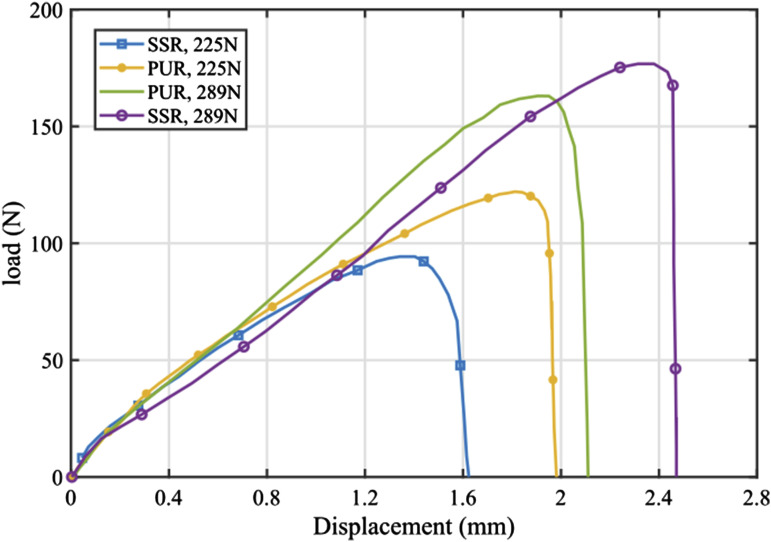
Comparison of load-displacement curves for uncured prepreg tapes bonded (SLJ) samples under different loads.

The load-displacement curves for SLJ samples created under the four different levels of pressure are shown in Figure 5. The most significant observations from Figure 5 can be outlined as: The elastic regions which are identified as the sections of the load-displacement curve with the steepest slope at the initial stage, indicate similar characteristics in all samples. The viscoelastic behavior of the interfacial bonding in the bonded prepreg tapes and the occurrence of cracks in the bonded area due to tension leads to a gradual decline in the slope of the force-displacement curve over time until it reaches the failure point. Because of the pressure sensitivity inherent in uncured thermoset prepreg, it is observed that an increase in load from 225 N to 289 N leads to a 33% and 86% increase in the failure load or PUR and SSR, respectively. Notably, the results indicate that at the lower force (225 N), although the pressure applied by PUR is less than that of SSR, the larger contact area for PUR results in a higher dwell time. This extended dwell time contributes to creating a stronger bond between the two prepreg tapes. However, at higher force (289 N), the greater pressure applied by SSR compensates for the difference in dwell time, leading to a significantly stronger bond between the two prepreg pieces.

### Feed rate and dwell time effects

**Figure 6.**
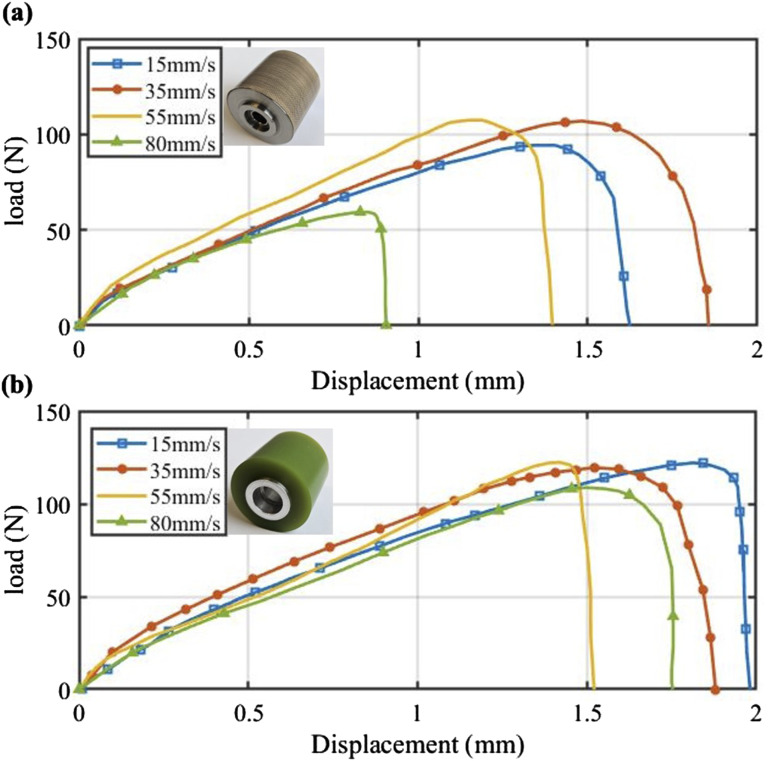
Comparison of load-displacement curves for SLJ specimens fabricated at 25°C and 225 N under three different feed rates and compacted by: (a) SSR and (b) PUR.

As mentioned, SLJ samples were produced at different lay-up speeds of 15 mm/s, 35 mm/s, and 50 mm/s. The resulting force-displacement curves are shown in Figure 6, demonstrating that PUR samples exhibit higher failure loads and strains to failure compared to SSR. For SSR, among the lay-up speeds of 15 mm/s, 35 mm/s, 50 mm/s, and 80 mm/s, the minimum failure load corresponds to the 80 mm/s speed. This is likely due to the high speed preventing the bonding from forming effectively. However, between 15 mm/s, 35 mm/s, and 50 mm/s, the minimum failure load occurs at 15 mm/s. This can be attributed to the reduced feed rate potentially leading to uneven fiber distribution or alignment, along with an increase in width and a decrease in thickness as the resin is pushed towards the sides. The influence of feed rate is more significant in SSR samples than in PUR ones. Increasing the feed rate from 15 mm/s to 35 mm/s and 50 mm/s in SSR leads to a 13% and 13.5% increase in failure load, respectively. However, similar speed increments in PUR samples result in nearly identical failure loads. This can be explained by the difference in dwell time, where PUR benefits from a longer dwell time compared to SSR, allowing the tape sufficient time to bond effectively.

The longer dwell time for PUR is directly related to its larger contact length. As shown in Figure 3, the contact length for SSR is only 1.5 mm, while the contact length for PUR is about 8 mm. This extended contact length in PUR provides better dwell time, even at higher speeds, which results in improved bonding. As a result, changes in bonding strength with varying speeds are less pronounced for PUR than for SSR.

### Temperature effect

**Figure 7.**
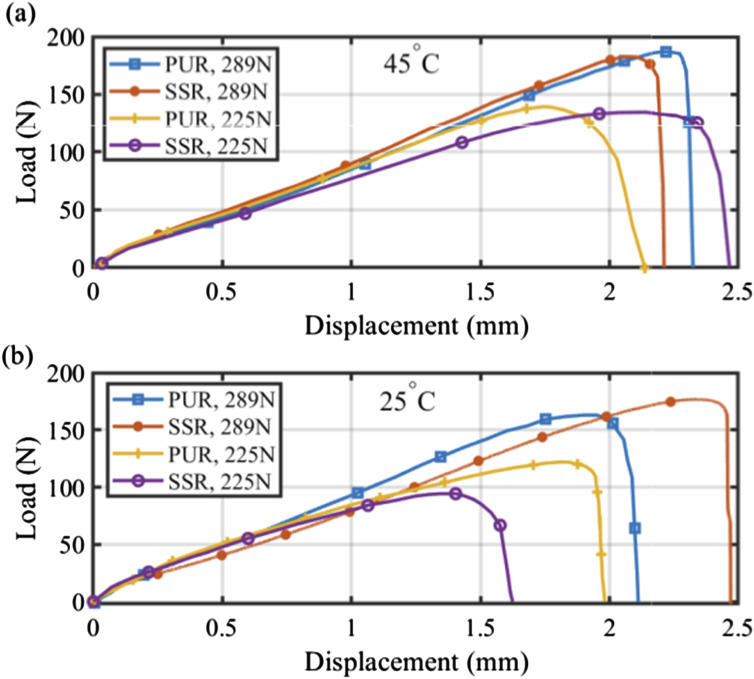
Comparison of load-displacement curves for SLJ specimens fabricated with the feed rate of 14 mm/s under different levels of pressures and temperatures: (a) 45°C and (b) 25°C.

The SLJ specimens were fabricated at two different temperature levels: (i) at room temperature, approximately 25 degrees Celsius, and (ii) at an elevated temperature of around 45 degrees Celsius. It is noteworthy that in the process of creating these single lap joint samples, the prepreg pieces underwent initial heating. Subsequently, the compaction roller was applied at various feed rates to ensure the bonding of all samples at the elevated temperature of 45 degrees Celsius. Figure 7 shows the load-displacement curves of SLJ samples at different temperature levels. These results illustrate that an increase in temperature from 25 to 45 degrees Celsius leads to a rise in shear strength in the bonded prepreg samples. This enhancement is ascribed to increasing resin diffusion at the bonded region. Indeed, the increased temperature reduces the resin viscosity which results in a rise in resin diffusion at the interface, thereby leading to the increased shear strength observed in the SLJ samples. Certainly! It’s essential to note that there’s an optimal temperature level, for temperatures below this threshold lead to high viscosity, preventing diffusion of resin in the inter-laminar layer, while temperatures above it can cause excessive flow or even curing of the resin in the bonding area during the lay-up process (before autoclave process) that both scenarios can have a negative impact the part’s quality. Besides, it can be observed that as the temperature increases, the failure load results for the two compaction rollers tend to become closer. Indeed, with the increasing temperature, the influence of pressure on modifying the failure load diminishes.

**Figure 8.**
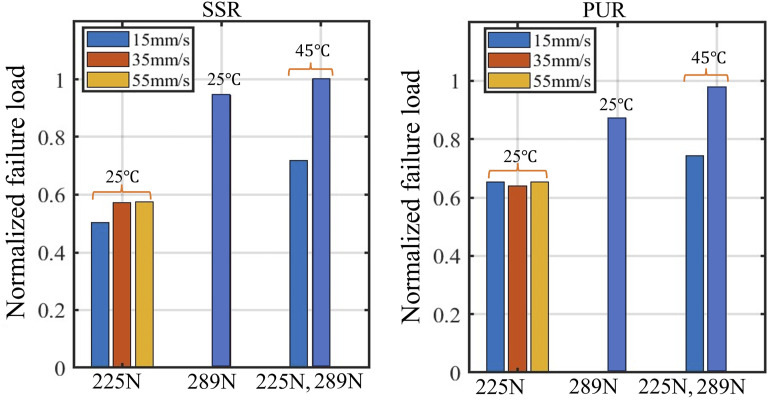
Comparison of experimentally obtained failure load of uncured prepreg SLJ samples created under different process conditions.

To gain a deeper insight into how pressure, temperature, and feed rate affect shear strength, we have represented the failure loads of SLJ samples shown in previous graphs as a bar chart in Figure 8. For ease of comparison, all failure loads have been normalized by dividing them by the maximum failure load, which corresponds to the sample created under the conditions of a temperature of 45 and a pressure of 289 N by SSR. Generally, it can be concluded that elevating both pressure and temperature to 289 N and 45°C, respectively, has a beneficial impact on enhancing inter-laminar strength, while simultaneously reducing the influence of dwell time on inter-laminar strength.

## Geometric and microscopic analysis

The results are presented in Table 2 provides a summary of the measurements conducted on the width and thickness of SLJ samples produced under various conditions. The measurements reveal that as the load and temperature rise, the width increases while the thickness decreases. Notably, this change is more significant for SSR than the PUR. Moreover, It’s important to note that excessively increasing the width may result in defects like gaps and overlaps during the process.

**Table 2.** Effect of process parameters on the geometry of the prepreg tape and SLJ overlap section.

Process conditions			Measurements					
Type of roller	Temperature and load	Feed-rate	Width (mm)	Standard deviation	Thickness (mm)	Standard deviation	Thickness of overlap (mm)	Standard deviation
PUR	T = 25C	15 mm/s	6.64	0.023	0.166	0.0013	0.322	0.0012
	L = 225N	35 mm/s	6.55	0.023	0.167	0.0016	0.323	0.0013
		55 mm/s	6.48	0.024	0.170	0.0018	0.334	0.0016
	T = 25C	15 mm/s	6.82	0.021	0.16	0.0012	0.321	0.0012
	L = 289N							
	T = 45C	15 mm/s	6.78	0.02	0.162	0.001	0.322	0.001
	L = 225N							
SSR	T = 25C	15 mm/s	6.87	0.033	0.153	0.0012	0.304	0.0014
	L = 225N	35 mm/s	6.85	0.04	0.153	0.0012	0.303	0.0012
		55 mm/s	6.81	0.052	0.156	0.0013	0.31	0.0011
	T = 25C	15 mm/s	6.97	0.021	0.151	0.0012	0.30	0.0013
	L = 289N							
	T = 45C	15 mm/s	6.95	0.02	0.151	0.001	0.301	0.0012
	L = 225N							

**Figure 9.**
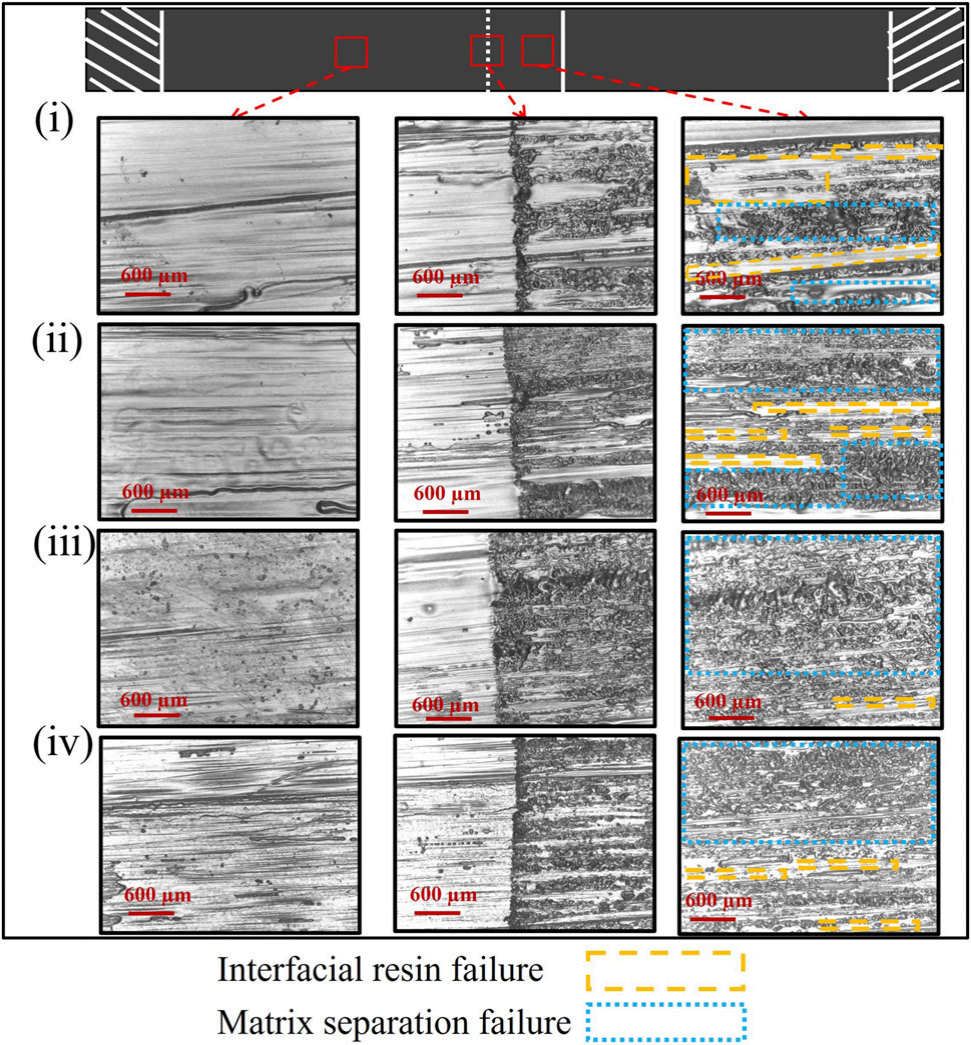
Captured micrographs of SLJ samples created with PUR under varied conditions: (i) 35 mm/s feed rate and 25°C and 225 N, (ii) 15 mm/s feed rate and 25°C and 225 N, (iii) 15 mm/s feed rate and 45°C and 225 N, (iv) 15 mm/s feed rate and 25°C and 289 N.

**Figure 10.**
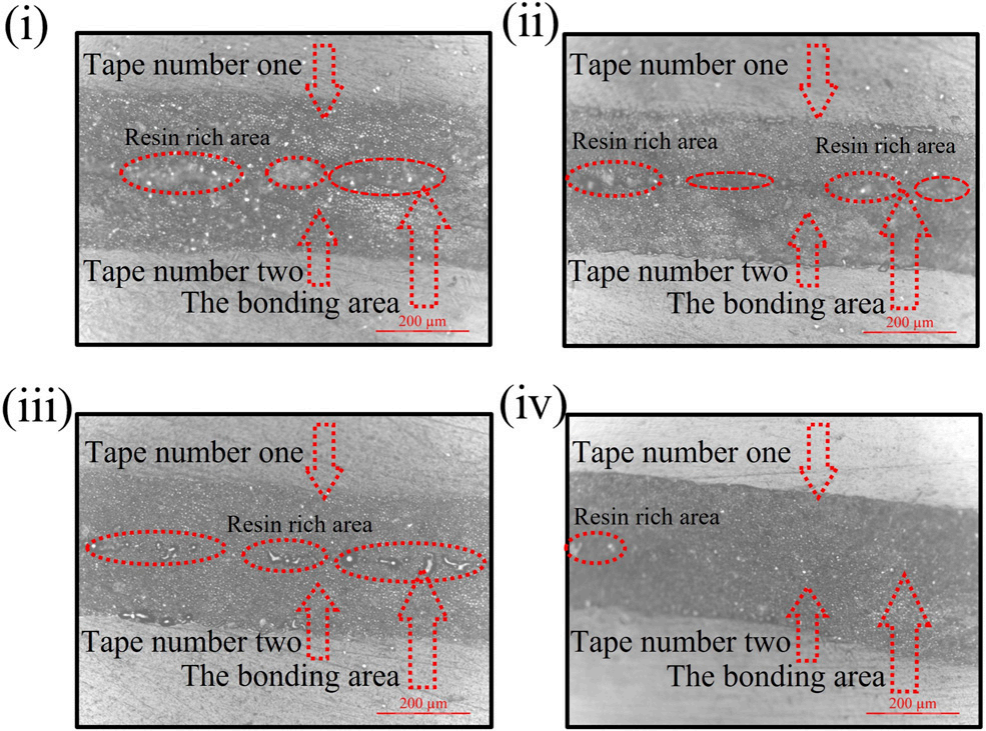
Captured micrographs of the cross-section of SLJ specimens in the bonding area before the tensile test fabricated with PUR under varied conditions: (i) 35 mm/s feed rate and 25°C and 225 N, (ii) 15 mm/s feed rate and 25°C and 225 N, (iii) 15 mm/s feed rate and 45°C and 225 N, (iv) 15 mm/s feed rate and 25°C and 289 N.

The photomicrographs are captured from the samples after the test using optical microscopy to illustrate catastrophic failures and bonding areas in SLJ samples created under varying conditions. The pictures are taken from three different regions of each sample: (1) in an unbounded area, (2) with half the frame showing the bonding area and the other half depicting anon-bonding area, and (3) exclusively from the bonding region. Figure 9 shows the micrographs taken from SLJ samples fabricated by PUR under four different conditions: (i) Temperature of 25°C, speed of 35 mm/s, and pressure of 225 N, (ii) Temperature of 25°C, speed of 15 mm/s, and pressure of 225 N, (iii) Temperature of 45°C, speed of 15 mm/s, and pressure of 225 N, (iv) Temperature of 25°C, speed of 15 mm/s, and pressure of 289 N. The analysis of these micro-graphs reveals several significant findings: The visuals indicate a mixed failure mode, involving both interfacial resin failure (analogous to adhesive failure in systems with a distinct adhesive layer) and matrix separation (analogous to cohesive failure in such systems) in the SLJ samples. The areas corresponding to interfacial resin failure and matrix separation are indicated in Figure 9 by yellow dashed lines and blue square-dotted lines, respectively. Notably, as the feed-rate decreases from 35 mm/s to 15 mm/s in samples produced with PUR, there is a noticeable enhancement in bonding. This is evidenced by a reduction in the interfacial resin failure region and a more pronounced occurrence of matrix separation, indicating an improved bonding quality. To further investigate the bonding quality, photomicrographs of the cross-sections of the same samples presented in Figure 9 were captured before conducting the tensile tests. Figure 10 displays these images, providing a detailed view of the bonding interface between the two uncured prepreg tapes. As shown in Figure 10, samples produced under conditions (i) and (ii) exhibit significant resin-rich regions at the bonding area, suggesting weak bonding between the prepreg tapes. Conversely, in specimens (iii) and (iv), the increased pressure and temperature resulted in a reduction of resin-rich areas, indicating an improved bonding quality between the prepreg tapes.

## Numerical simulation and results

### Description of the FE model

The ABAQUS/Explicit software was considered for this research to model the mechanical behavior of the SLJ samples subjected to the quasi-static tensile loading. One of the common methodologies for simulating the interface layer is the cohesive zone model (CZM). This model is characterized based on a traction-separation law, which defines the correlation between the stress (traction) applied across the interface and the corresponding displacement.^23,24^ In this context, there is extensive literature on modeling the mode I and II fracture of composite laminates through different approaches of the cohesive zone model.^25–28^ The stickiness of uncured prepreg tapes to the substrate was modeled during the steering process by employing the cohesive zone model in Ref. 29. Therefore, in this research, a cohesive zone model is developed for the simulation of the SLJ behavior of the prepreg tapes bonded.

In this approach, the force-displacement curves obtained from the SLJ tests are first used to generate the traction-separation (T-S) curves. These T-S curves are then utilized to define the properties of the cohesive element. According to the force-displacement curves shown in the experimental results section, it is evident that the T-S curves exhibit a non-linear softening part due to the damage propagation in the bonded interface. The relationship between traction and separation can be defined in the form of:

(2) $$T_{t}=K_{t}\delta_{t}$$

where *T*_*t*_ and *δ*_*t*_ represent the traction and relative displacement in the shear direction, respectively. *K*_*t*_ is the stiffness parameter of the interface layer is expressed as:

(3) $$K_{t}=\left\{\begin{array}{c} K_{0}\;if\;\delta \leq \delta_{0}\\ K_{0}\left(1-D_{t}\right)\;if\;\delta_{0}<\delta <\delta_{f}\\ 0\;if\;\delta \geq \delta_{f} \end{array}\right.$$

where *δ*, *δ*_0_, and *δ*_*f*_ are the separation (displacement) in the shear direction, the amount of separation as far as the elastic region exists (before the softening region), and the separation corresponding to complete damage. *K*_0_ is the stiffness of the elastic region and the parameter *D*_*t*_ is a damage parameter, representing the ratio of the actual stress obtained from the test to the stress that would occur if there were no damage in the interface layer. This parameter can be determined using the following formula:

(4) $$D_{t}=1-\frac{T_{t}}{K_{0}\delta }$$

**Figure 11.**
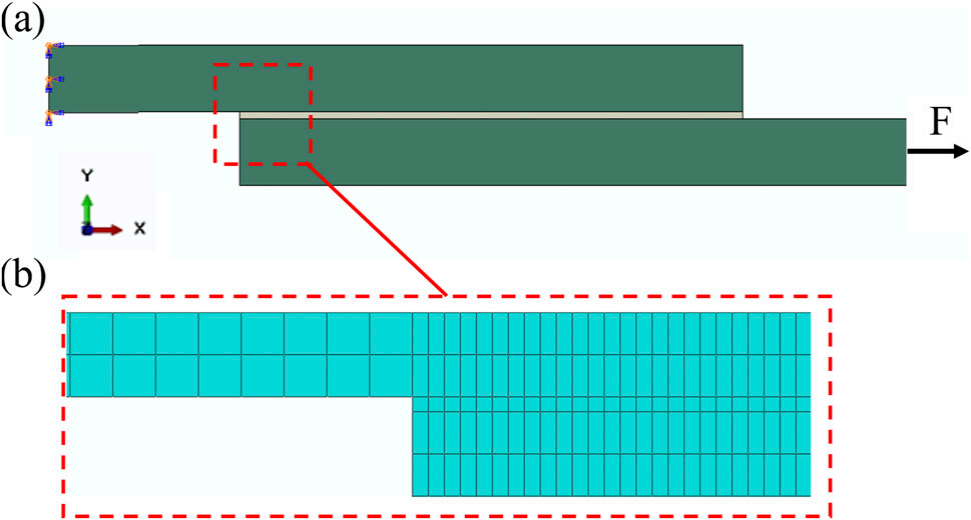
FE model of the single lap joint: (a) configuration, and boundary conditions, (b) mesh details.

Based on equations (4) and (3), when *D*_*t*_ = 0 it indicates that no damage has occurred, and when *D*_*t*_ = 1, it denotes that failure has occurred. Therefore, to accurately define the cohesive element properties for the bonding between the prepreg tapes into the Abaqus software, two parameters are needed: elastic parameters and damage evolution parameters. Elastic parameters are derived from the linear elastic region of the traction-separation curve, while damage evolution parameters are extracted from the softening region of the T-S curve and input into Abaqus in the tabular form as a function of displacement (separation). In our simulation, prepreg tapes as adherents of the SLJ samples are modeled using the mechanical properties presented in Table 3 and they are meshed using solid elements, specifically the 8-node (C3D8R) elements. The interface layer at the bonding area is also meshed using an 8-node three-dimensional cohesive element (COH3D8). The shape and final meshed model of the SLJ sample are depicted in Figure 11.

**Table 3.** Mechanical properties of prepreg tape as an adherend for the SLJ simulation.^5^

*E*_1_ (GPa)	*E*_2_ (MPa)	*E*_3_ (MPa)	*G*_12_ (MPa)	*ν*_12_
120	0.046	0.046	3.025	0.2

### Simulation results

**Figure 12.**
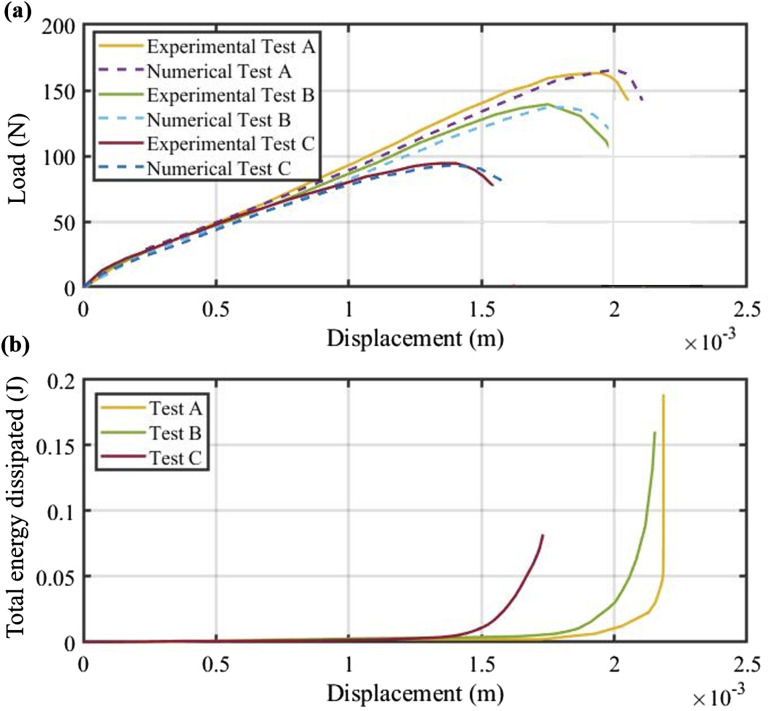
Verification and total energy dissipated by damage based on the FE model: (a) Comparison between the experimental and numerical load-displacement results. (b) Total energy dissipated by damage in each specimen.

To assess the simulation model developed in this study, the characteristics of the SLJ samples generated under specific process conditions were input into the Abaqus software. In order to verify the accuracy of the model, Figure 12(a) presents the force-displacement curves, both numerical and experimental, for three specific tests within the experimental set. Test A reveals results for a sample produced at 289N, 14 mm/s, and 25°C using PUR. Test B displays results for a sample manufactured at 225N, 14 mm/s, and 45°C using PUR. Lastly, Test C shows the results for a sample created at 225N, 14 mm/s, and 25°C using SSR. The outcomes from the simulation reveal an excellent agreement between the model and experimental findings. One valuable factor in understanding inter-laminar shear behavior is the energy dissipated by damage. The FE simulation can capture this energy, as depicted in Figure 12(b). Initially, when there is no damage in the specimen (elastic region), the energy dissipated by damage remains zero. As displacement increases the energy dissipated shows a gradual increase, and after surpassing the maximum force the damage energy dissipated in the specimen sharply rises. This increase shows damage propagation in the interface layer of SLJ samples.

**Figure 13.**
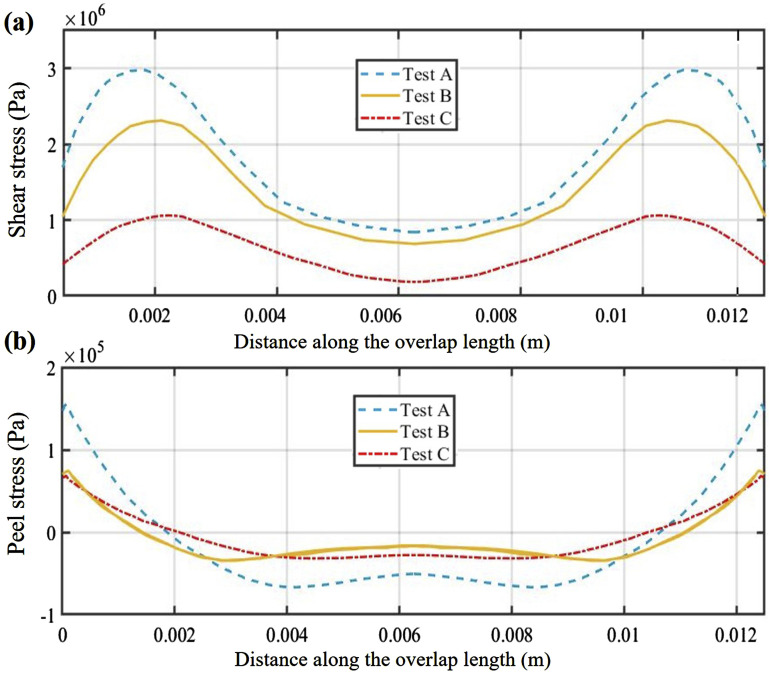
Adhesive shear and peel stress distribution for uncured prepreg SLJ samples: (a) adhesive shear distribution, (b) peel stress distribution at the overlap area.

Having validated the integrity of the developed model, the simulation can be employed in enhancing our comprehension of the shear behavior of SLJ samples. The shear stress distribution in the sample is shown in Figure 13, where it becomes evident that the highest shear stress values are concentrated at the regions near corners of the overlap, and by moving towards the center of the overlap, these stress values gradually decrease. Moreover, due to the load eccentricity in SLJ, there will be a bending moment and normal (peel) stress at the sample with the highest levels occurring at the corners (see Figure 13(b)). This indicates why the initiation of inter-laminar damage primarily originates from these corner areas.

**Figure 14.**
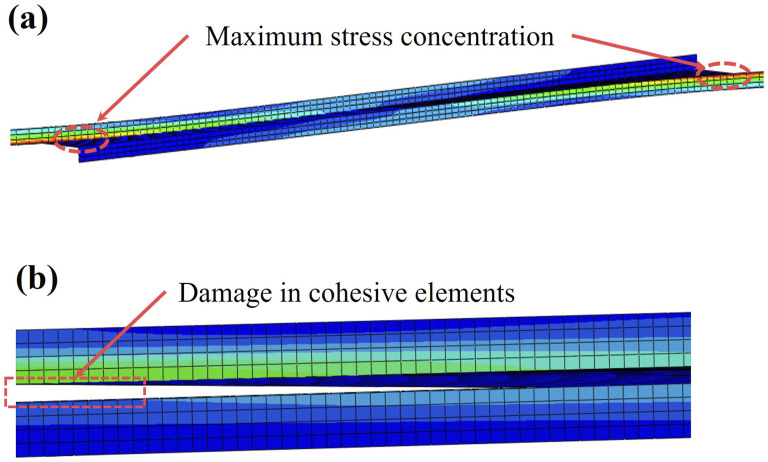
The results of the SLJ FE model: (a) stress concentration in SLJ samples before failure, (b) damage initiation in cohesive elements.

In this context, Figure 14(a) shows the stress concentration in the corner of the prepreg SLJ samples prior to the onset of damage. Figure 14(b) shows the damage initiation in SLJ samples, where it is observed that cohesive elements near the corner have been eliminated due to exceeding their capacity, while cohesive elements near the center of the overlap continue to withstand the force applied to the SLJ sample.

## Conclusion

In this work, a comprehensive series of experimental and numerical investigations were conducted to deepen our understanding of the inter-laminar shear behavior of the uncured prepreg during the AFP process. To assess the shear response of the uncured prepreg, we utilize the single-lap bonded joint samples made of uncured prepreg tapes. These specimens were created, considering different levels of AFP process parameters including temperature, pressure, dwell time, and feed rate. This study, incorporating experimental and numerical approaches, pursues two main goals: predicting optimal parameters for automated fiber placement (AFP) to maximize inter-laminar shear strength during the AFP process and developing a shear strength model for uncured prepreg bonding to find the distribution of the stresses in the interfacial bonding along the overlap length.

The most important findings by analyzing the experimental data are summarized as:

• Generally, increasing the temperature from 25°C to 45°C and raising the load applied on the rollers from 225 to 289 leads to an improvement in inter-laminar shear strength by enhancing the bond between the two prepreg tapes within the overlap range. In this context, increasing the load has a more pronounced impact on enhancing the shear strength of samples produced using SSR compared to PUR. Meanwhile, elevating the temperature exerts a more significant influence on improving the shear strength of samples fabricated by PUR.
• A significant observation derived from the shear strength results of samples produced by two rollers under a lower load of 225 N is that, despite the SSR applying greater pressure to the samples compared to the PUR, the shear strength of samples created by the PUR surpasses that of the SSR. This is attributed to the extended dwell time or compaction period of the PUR. However, upon increasing the load on the rollers to 289, the pressure applied by the SSR significantly exceeds that of the PUR (See Figure 4). This heightened pressure diminishes the impact of the compaction period, resulting in well-formed bonding in the samples produced by the SSR, even with a shorter compaction period.
• While there are overall conclusions regarding the effect of parameters on shear strength, the experiment results imply that the prediction of the shear strength of SLJ samples is not exclusively reliant on individual parameters. Rather, it is connected to the combination of various process parameters. Indeed, it is not straightforward to determine that changes in each parameter lead to specific outcomes. Instead, it is urgent to consider all parameters together for deriving meaningful conclusions regarding the shear strength of the samples.

Furthermore, micrograph images of the PUR indicate a mixed failure mode in SLJ samples produced from prepreg tapes. In addition, it was observed that in the process of creating SLJ samples with the PUR, improving bonding quality is achieved by reducing the feed rate and increasing the temperature and pressure from 25°C to 45°C and from 225 N to 289 N, respectively. Remarkably, the temperature and pressure increase has a more pronounced effect on bonding quality compared to the feed rate, likely because higher temperatures reduce the viscosity of the resin, leading to a more extensive resin diffusion at the bonded area. Finally, a FEM model was developed which is demonstrating excellent agreement with experimental findings. Consequently, the FEM model proved valuable for assessing the complex stress distribution within the adhesive layer and adherents under various Automated Fiber Placement (AFP) process conditions. Additionally, it provided insights into the damage behavior occurring in the interface layer of bonded prepreg tapes.

## Data Availability

Data sharing not applicable to this article as no datasets were generated or analyzed during the current study.
